# Wnt5a Increases Cardiac Gene Expressions of Cultured Human Circulating Progenitor Cells via a PKC Delta Activation

**DOI:** 10.1371/journal.pone.0005765

**Published:** 2009-06-02

**Authors:** Masamichi Koyanagi, Masayoshi Iwasaki, Judith Haendeler, Michael Leitges, Andreas M. Zeiher, Stefanie Dimmeler

**Affiliations:** 1 Institute of Cardiovascular Regeneration, Center for Molecular Medicine, J.W. Goethe University, Frankfurt, Germany; 2 The Biotechnology Centre of Oslo, University of Oslo, Oslo, Norway; 3 Department of Cardiology, Internal Medicine III, J.W. Goethe University, Frankfurt, Germany; University of Cincinnati, United States of America

## Abstract

**Background:**

Wnt signaling controls the balance between stem cell proliferation and differentiation and body patterning throughout development. Previous data demonstrated that non-canonical Wnts (Wnt5a, Wnt11) increased cardiac gene expression of circulating endothelial progenitor cells (EPC) and bone marrow-derived stem cells cultured in vitro. Since previous studies suggested a contribution of the protein kinase C (PKC) family to the Wnt5a-induced signalling, we investigated which PKC isoforms are activated by non-canonical Wnt5a in human EPC.

**Methodology/Principal Findings:**

Immunoblot experiments demonstrated that Wnt5a selectively activated the novel PKC isoform, PKC delta, as evidenced by phosphorylation and translocation. In contrast, the classical Ca^2+^-dependent PKC isoforms, PKC alpha and beta2, and one of the other novel PKC isoforms, PKC epsilon, were not activated by Wnt5a. The PKC delta inhibitor rottlerin significantly blocked co-culture-induced cardiac differentiation in vitro, whereas inhibitors directed against the classical Ca^2+^-dependent PKC isoforms or a PKC epsilon-inhibitory peptide did not block cardiac differentiation. In accordance, EPC derived from PKC delta heterozygous mice exhibited a significant reduction of Wnt5a-induced cardiac gene expression compared to wild type mice derived EPC.

**Conclusions/Significance:**

These data indicate that Wnt5a enhances cardiac gene expressions of EPC via an activation of PKC delta.

## Introduction

Various different types of adult stem or progenitor cells were shown to express cardiac genes and acquire a cardiac phenotype, when exposed to a cardiogenic environment. However, the incidence of cardiac differentiation is rather low in several studies, and it is unclear to what extent a fully functional maturation can be achieved. Therefore, the elucidation of signaling pathways controlling cardiac differentiation is of major importance to improve cardiac gene expression and functional maturation.

Wnt proteins are well known as crucial signaling molecules involved in physiological development [1], cancer development, as well as decision of stem cell fate [2]. Wnts play an important role for self renewal of hematopoietic stem cells [3] and maintenance of pluripotency of mouse embryonic stem cells [4]. Wnt5a promotes epithelial-to-mesenchymal transition in a melanoma cell line [5]. With respect to cardiovascular development and differentiation, both canonical and non-canonical Wnt signalings are important regulators. Inhibition of canonical Wnt signaling induces heart formation [6]. On the other hand, canonical Wnt signaling via GSK3β and β-catenin contributed to cardiac differentiation of mouse P19 cells [7], and Isl1^+^ cardiac progenitors [8]. For cardiac differentiation of embryonic stem cells, canonical Wnt/ß-catenin signaling is essential during early stages, but it inhibits cardiogenesis at later time points [9]. Non-canonical Wnts comprise Wnt-4, -5 and -11. As shown in model organisms, Wnt-11 stimulates cardiogenesis [10]. Moreover, Wnt-11 was shown to increase cardiac gene expressions in EPC and bone marrow derived mesenchymal stem cells [11], [12], and Wnt-11 induced cardiomyogenic differentiation in unfractionated bone marrow mononuclear cells [13]. Non-canonical Wnt signaling also enhances differentiation of Sca1^+^/c-kit^+^ adipose-derived murine stromal cells into spontaneously beating cardiomyocytes [14]. These data suggest that non-canonical Wnts such as Wnt5a and Wnt11 might be interesting candidates to enhance cardiac gene expression in adult progenitor cells.

In contrast to the well defined ß-catenin-dependent canonical Wnt signaling, the pathways mediating non-canonical Wnt signaling are not fully understood. Several different non-canonical Wnt pathways were proposed, including calcium-dependent signaling, the planner cell polarity (PCP) pathway via activation of the Rho kinase, activation of the c-Jun N-terminal kinase (JNK), or protein kinase C (PKC) [15]. With respect to cardiac differentiation in adult progenitor cells, several studies showed that non-canonical Wnts induce cardiac gene expression in a PKC-dependent manner [11]–[13]. However, the PKC isoforms have not been identified.

Using both, pharmacological inhibitor as well as genetic ablation in vivo, we identify the novel PKC isoform, PKC delta, to importantly contribute to cardiac gene expression in EPC, indicating that PKC delta is a key target of the non-canonical Wnt pathway.

## Results

### Addition of Wnt5a increased cardiac gene expression

**Figure 1:**
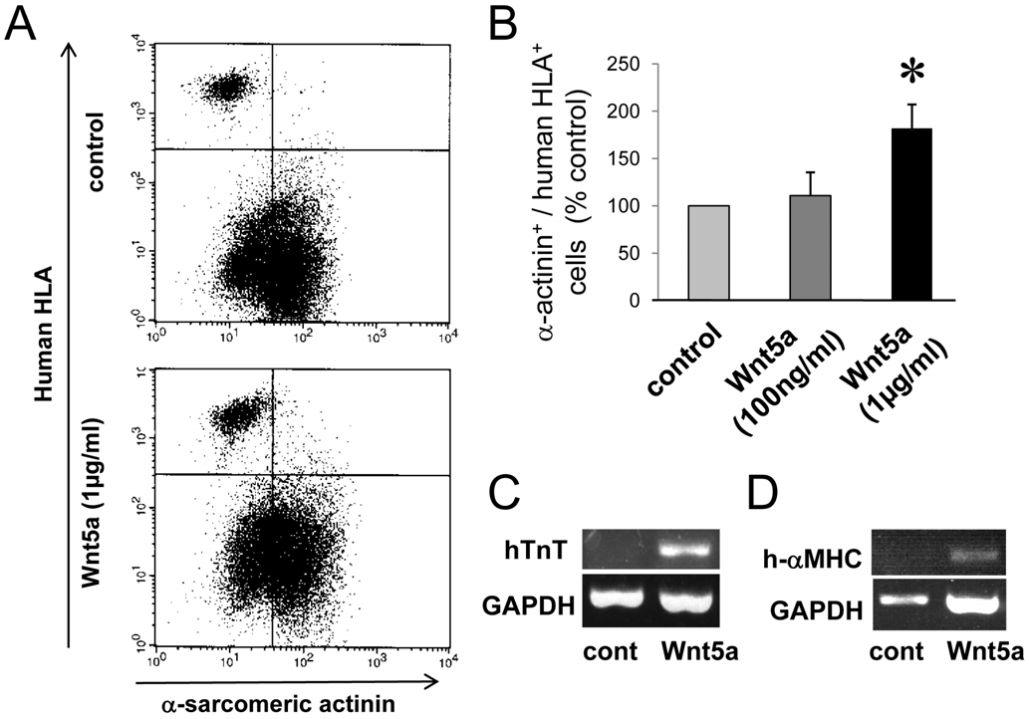
Wnt5a increased cardiac differentiation of EPC. EPCs were co-cultured with rat cardiomyocytes and analyzed after 6 days. (A) Representative flow cytometry analysis using human HLA and alpha sarcomeric actinin. (B) Summary of dose dependent Wnt5a (100 ng/ml or 1 μg/ml) effects on human HLA and alpha sarcomeric actinin double positive cells. n = 5. * indicates p<0.05 vs control. (C/D). RT-PCR images of human specific troponin T (hTnT), alpha myosin heavy chain (h-αMHC), or GAPDH in control or Wnt5a treated cells.

First, we investigated the effect of two different doses of Wnt5a (100 ng/ml and 1 μg/ml) on cardiac gene expression of EPC co-cultured with neonatal rat CMs. Wnt5a at a concentration of 1 μg/ml significantly increased the number of cells positive for human HLA and alpha-sarcomeric actinin, which represent human cells expressing cardiac genes (Fig. 1A/B). The enhancement of cardiac gene expression was further confirmed by RT-PCR using human-specific primers selectively detecting human troponin T and human alpha-myosin heavy chain. The mRNA-expression of both genes was increased by Wnt5a treatment (Fig. 1C/D). To confirm that the PCR product indeed represents human troponinT, we sub-cloned the human-troponin T PCR products using pGEM-Teasy vector and analyzed the sequences. Sequences were 100% identical to human troponin T (supplementary figure S1A).

### Wnt5a activates PKC delta but not PKC epsilon

**Figure 2:**
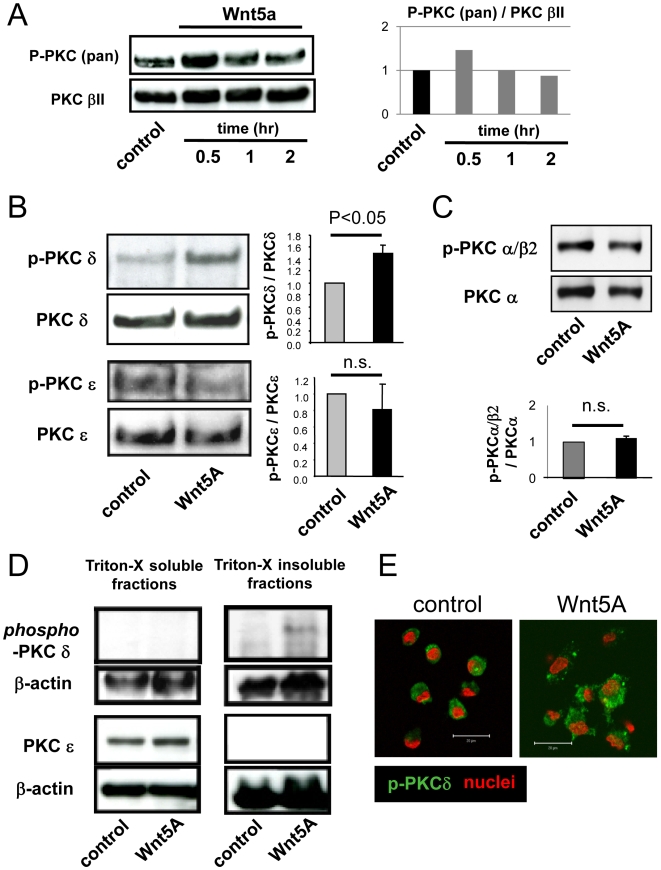
PKC activation by Wnt5a. PBS or Wnt5a (1 μg/ml) was incubated for 30 minutes in all experiments. (A) Time dependent phosphorylation of pan-PKC after treatment with Wnt5a (1 μg/ml). (B) Representative phospho-PKC delta (p-PKCδ), PKCdelta (PKCδ), phospho-PKC epsilon (p-PKCε), and PKCepsilon (PKCε) and summaries of the quantification are shown. n = 3. (C) Representative phospho-PKC alpha/beta2, PKCalpha, PKCbeta2, and summary of data are shown. n = 3. (D) Translocations of phospho-PKC delta and PKC epsilon. Representative immunoblots of triton soluble and insoluble fractions are shown. n = 3–4. (E) Immunochemistry of phospho-PKC delta. Green indicates phospho-PKC delta. Red indicates nuclei.

According to our previous data, cardiac gene expression in EPC was inhibited by pan-PKC inhibitors, Bisindolylmaleimide I and Bisindolylmaleimide III [11], whereas the PKC activator phorbol-12-myristate-13-acetate (PMA) increased cardiac gene expression [11]. These data excluded the involvement of PMA-independent atypical PKC isoforms. Therefore, we investigated the potential involvement of calcium-dependent classical PKC or novel PKC isoforms to mediate Wnt5a-induced increases of cardiac gene expression. First, we investigated which PKC isoforms are activated by Wnt5a in EPC. Since pan phospho-PKC was increased 30 minutes after incubation with Wnt5a (Fig. 2A), we stimulated cells with Wnt5a for 30 minutes. As shown in figure 2B, phosphorylation of PKC delta, which is a novel PKC isoform, was significantly increased by Wnt5a, whereas phosphorylation of another novel PKC, PKC epsilon, and the calcium-dependent classical PKCs (PKC alpha and beta2) did not change (Fig. 2B/C). These data suggested that PKC delta appears to be selectively activated by Wnt5a. In order to corroborate the selectively activation of PKC delta by Wnt5a, we examined the translocation of PKC delta and PKC epsilon to the triton X-insoluble fraction. Since total PKC delta level was high in the triton X-soluble and -insoluble fraction without stimulation of Wnt5a (data not shown), we decided to use phospho-PKC delta antibodies to detect the active form of PKC delta. Phosphorylation of PKC delta was only detected in the triton X-insoluble fraction after treatment with Wnt5a, indicating that the active form of PKC delta was present in the cytoskeleton (triton X-insoluble) fraction (Fig. 2D). In accordance, immunochemistry revealed that phospho-PKC delta can be detected in association with the membrane and cytoskelton (Fig. 2E). Interestingly, only 30 minutes stimulation with Wnt5a, the morphology of EPCs was clearly changed (Fig. 2E). Taken together, these data indicate that Wnt5a selectively activates the PKC delta isoform in EPC.

### Cardiac gene expression is attenuated by inhibition of PKC delta

**Figure 3:**
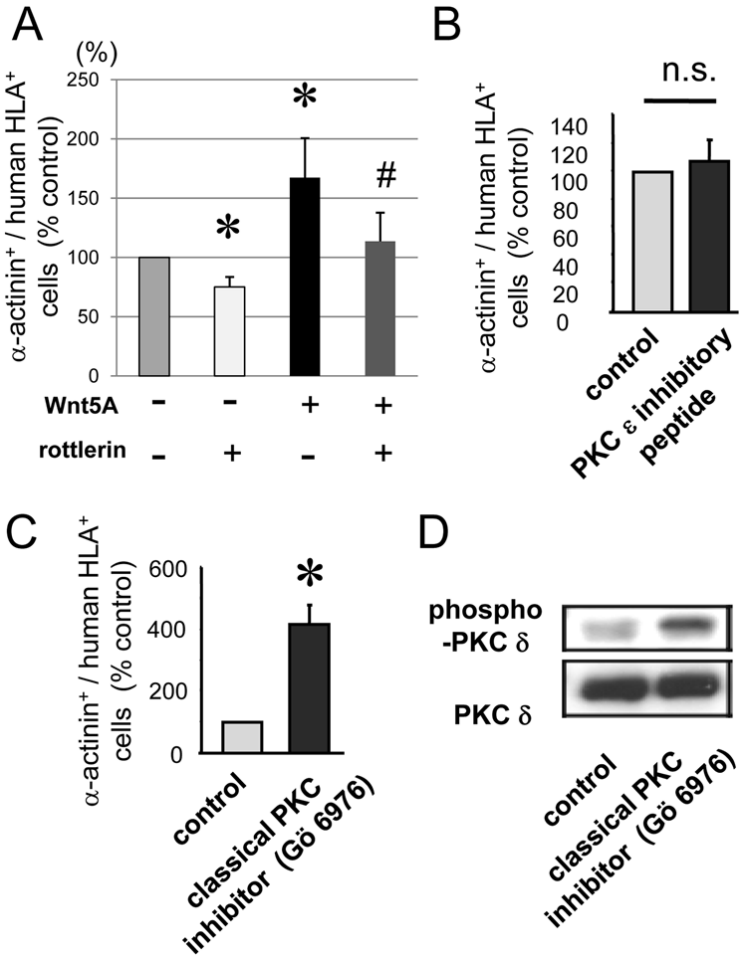
The effects of pharmacological PKC inhibitors on cardiac differentiation and phosphorylation. (A–C) EPCs from human peripheral blood were co-cultured with rat cardiomyocytes (CM) and analyzed after 6 days. (A) Summary of cardiac differentiation which are demonstrated by flow cytometry analysis. Human HLA and alpha sarcomeric actinin double positive cells were counted. Wnt5a (1 μg/ml) or PKC delta inhibitor, rottlerin (1 μM) were used. * indicates p<0.05 vs control without Wnt5a and rottlerin. # indicates p<0.05 vs Wnt5a without rottlerin. n = 5–7. (B/C) PKC epsilon inhibitory peptide (B) or classical PKC inhibitor (C; Gö 6976) were used. * indicates p<0.05 vs control. n = 5–6. (D) Phosphorylation of PKC delta in control (PBS) and Gö 6976 treatment (both 30 min.) are shown. PKC delta served as control.

Finally, we determined the role of PKC delta for EPC cardiac gene expression. Incubation with the relatively selective PKC delta inhibitor, rottlerin (1 μM), significantly attenuated cardiac differentiation (Fig. 3A). Importantly, increased cardiac marker gene expression by Wnt5a was also inhibited by rottlerin, suggesting that PKC delta is an important mediator for cardiac gene expression via non-canonical Wnt signaling. In contrast to the effect of PKC delta inhibition, PKC epsilon inhibition had no effect on cardiac gene expression (Fig. 3B). Surprisingly, incubation with the calcium-dependent PKC inhibitor, Gö6976, also increased cardiac gene expression (Fig. 3C). Gö 6976 also enhanced phosphorylation of PKC delta (Fig. 3D), suggesting that inhibition of the calcium-dependent PKC by Gö6976 is paralleled by an activation of PKC delta, which was likely contributing to increased cardiac gene expression.

**Figure 4:**
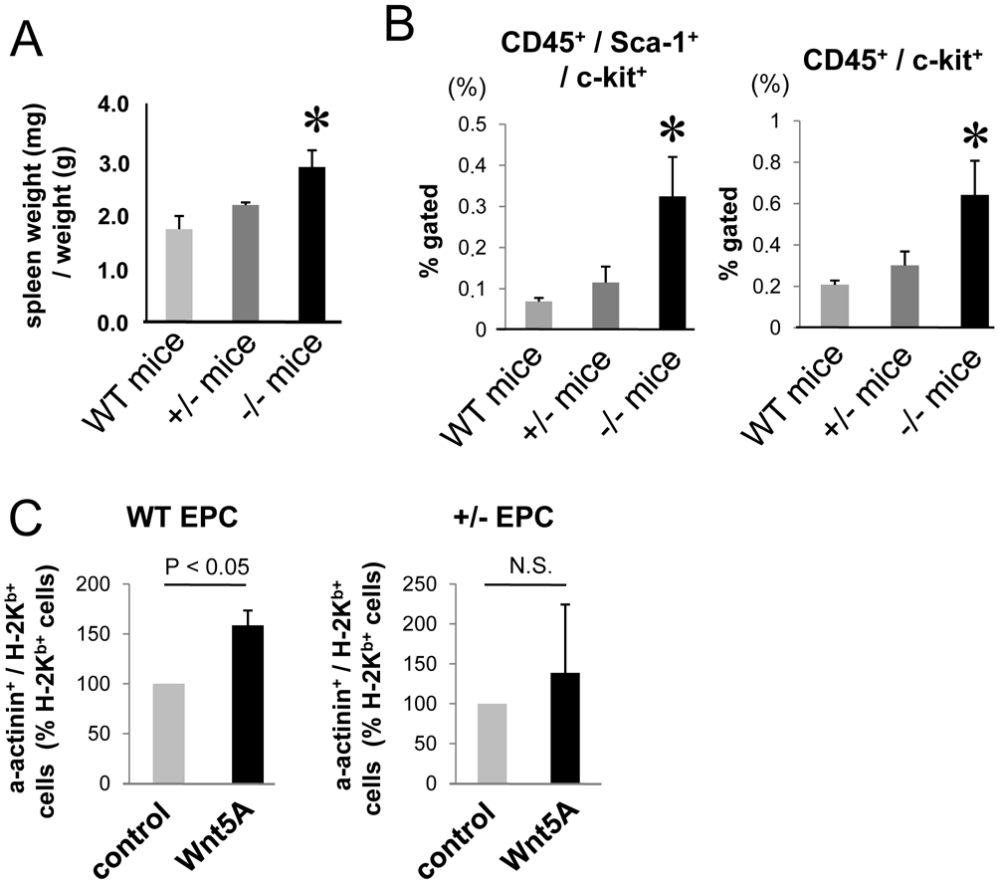
The characteristics and the effects of EPC isolated from PKC deficient mice. (A) Spleen weight per body weight in wild type (WT), PKC delta +/−, and PKC delta −/− mice. * indicates p<0.05 vs wild type mice. n = 3–4. (B) CD45+/Sca-1+/c-kit+ cells and CD45+/ c-kit+ cells in splenic mononuclear cells are shown. * indicates p<0.05 vs wild type mice. n = 3–4. (C) Mice EPC from wild type mice and PKC delta +/– mice were isolated from spleen and used for co-culture with rat cardiomyocytes with or without treatment with Wnt5a. Mouse MHC class I, H-2K^b^ was quantified by flow cytometry. n = 3–4.

In order to avoid potential unspecific effects of pharmacological PKC delta inhibition, we isolated EPC from the spleen of PKC delta deficient mice or wild type mice. Before investigating the cardiac differentiation capacity of splenic EPC, we characterized the mononuclear cells isolated from spleen in PKC delta −/− mice, PKC delta +/− mice, and wild type mice. The spleen size and several subsets of progenitor cell numbers (CD45^+^/c-kit^+^ cells and CD45^+^/sca-1^+^/c-kit^+^ cells) were significantly increased in PKC delta −/−, but not PKC delta +/− mice (Fig. 4AB). Furthermore, Wnt5a treatment significantly increased EPC number in PKC delta −/− mice, but had no effect on EPC numbers when EPC had been isolated of PKC delta +/− mice (data not shown). Since the characteristic and number of PKC delta −/− EPC is different from wild type or PKC delta +/− EPC, we purposely used spleen-derived EPC from PKC delta +/− mice, which showed normal hematopoietic stem cell numbers to perform the co-culture assay with neonatal rat CM. Wnt5a treatment only increased cardiac differentiation in EPC isolated from wild type mice, but not in EPC isolated from PKC delta +/− mice (Fig. 4C).

## Discussion

Both canonical and non-canonical Wnt signaling were shown to be important for cardiovascular development and differentiation. In particular, non-canonical Wnt pathways play a crucial role in cardiogenesis [10]. Protein kinase C is one of the key targets of non-canonical Wnt signaling. The importance of the Wnt/PKC pathway for cardiac gene expression was previously demonstrated in several adult stem cells [11]–[13] by using spontaneous differentiation assay [13] and co-culture systems [11], [12], [19]. We used a co-culture assay with rat CMs to mimic the cardiac environment in vitro in this study, because this assay is established and suitable to assess cardiac gene expression and the underlying signaling in EPC [12], [20]–[22]. The major finding of the present study is that a novel PKC isoform, PKC delta, is selectively activated by non-canonical Wnt5a and contributes to cardiac gene expression.

Protein kinase C is subdivided into 3 different groups: calcium-dependent diacylglycerol (DAG)-activated classical PKC (e.g. alpha, beta1, and beta2), calcium-independent DAG-activated novel PKC (e.g. delta and epsilon), and calcium-independent DAG-non-resposive atypical PKC (e.g. zeta and iota). Several PKC isoforms were reported to be activated in cellular differentiation processes. PKC epsilon isoform regulates the megakaryocytic differentiation of human CD34 cells [16] and induces astrocytic differentiation of neural precursor cells [17]. Differentiation of embryonic stem cells into beating cardiomyocytes is dependent on downregulation of PKC beta and zeta in concert with upregulation of PKC epsilon [18]. However, the role of PKC isoforms for cardiac differentiation in adult stem cells has not been well elucidated. Our data are the first to demonstrate the contribution of the PKC delta isoform to non-canonical Wnt signaling induced cardiac differentiation of adult progenitor cells.

PKC delta is a serine/threonine kinase and has important roles in growth regulation, tissue remodeling [19] and apoptosis [20]. PKC delta deficient mice develop almost normally and are fertile, but show abnormalities of the immune system [21]. In particular, PKC delta−/− mice have increased proliferation of B lymphocytes and lymphocytic organ infiltration [21]. Indeed, in the present study, we observed increases in spleen size and in the number of myeloid progenitor cell fractions (c-kit^+^ cells and sca-1^+^c-kit^+^ cells in CD45 fraction) within the splenic mononuclear cell population in PKC delta −/− mice (Fig. 4A/B). The increased number of sca-1^+^c-kit^+^ cells may be a consequence of a negative feedback of dysfunction of progenitor or stem cells in PKC delta-deficient mice. Therefore, in order to exclude any confounding secondary effects, we purposely used spleen-derived EPC from heterozygote mice, which showed normal hematopoietic stem cell numbers. Consistent with the data using pharmacological inhibitors, EPC derived from PKC delta +/− mice exhibited a reduced capacity to differentiate to CMs upon co-culture with neonatal CMs. Thus, these data suggest that PKC delta importantly contributes to cardiac differentiation of adult progenitor cells.

However, the signals downstream of Wnt5a-activated PKC delta signaling remain unclear. Preliminary experiments demonstrate that Wnt5a induces the gene expression of several epigenetic modulators, but does not directly increase sarcomeric proteins or endothelial proteins (RT-PCR, data not shown), suggesting that Wnt5a does not directly increase cardiac or endothelial gene expression but facilitates differentiation by modulation of the epigenetic control pathways. This notion is further supported by the finding that Wnt5a does not induce cardiac differentiation in EPC without co-culture with cardiomyocytes indicating that factors secreted by the neonatal myocytes or cell-to-cell contact are required in addition to non-canonical Wnt activation to induce cardiac marker gene expression. Further experiments are necessary to address this question.

In summary, our data identify PKC delta as a key target of the non-canonical Wnt signaling pathway contributing to cardiac gene expressions in EPC.

## Materials and Methods

### Cell Culture Experiments

EPCs were cultivated from human peripheral blood mononuclear cells as described [22]. In brief, total mononuclear cells (MNCs) were isolated from blood by density gradient centrifugation with Biocoll separating solution (Biochrom AG). MNCs were plated in endothelial basal medium (EBM) (CellSystems), with supplements (1 μg/ml hydrocortisone, 3 μg/ml bovine brain extract, 30 μg/ml gentamicin, 50 μg/ml amphotericin B, 10 μg/ml EGF), and 20% fetal calf serum on culture dishes coated with human fibronectin (Sigma). After 3 days in culture, adherent cells were used for experiments. Neonatal ventricular cardiomyocytes were isolated from 0–1 day old Wistar rats and cultivated as previously described [11]. Noncardiomyocytes (primarily fibroblasts) were separated from the cardiomyocytes by differential plating onto plastic dishes. Then EPCs (1.5×10^5^) and freshly isolated cardiomyocytes were plated onto gelatine coated dishes at a ratio of 1:3. Medium was changed every 2 days. Recombinant mouse Wnt5a (1 μg/ml, R&D) was used to stimulate cells. For the inhibitory assay, rottlerin (1 μM, Calbiochem), PKC epsilon translocation inhibitor peptide and its control peptide (both 25 μM, Calbiochem), or Gö6976 (100 nM, Calbiochem) were used.

Mice mononuclear cells were isolated by density gradient centrifugation from spleen extracts as described [23]. 12–16 week SV129 mice or PKC delta heterozygous (+/−), and homozygous (−/−) deficient mice were used [24]. Isolated mononuclear cells were plated in the same medium as human cells.

### Flow cytometry analysis and immunostaining

After 6 days of the co-culture, cells were stained with phycoerythrin-conjugated antibodies recognizing human HLA-DR and HLA-class I (both Caltag Laboratories), or mouse MHC class I (H-2K^b^, BD) followed by permeabilization using the Cytofix/Cytoperm kit (BD Pharmingen) and staining with FITC-conjugated (Pierce) anti-α-sarcomeric actinin antibody (clone EA-53, Sigma) as descrived [12], [19]. Cells were analyzed on a BD FACS Calibur cell sorter or BD FACS Canto (both BD Biosciences).

For immunostaining, cells were fixed with 4% paraformaldehyde for 15 minutes. After permeabilization with 0.2% saponin (Sigma), cells were incubated with anti-phospho-PKC delta (Santa Cruz), followed by staining with Alexa Flour 488 anti-goat IgG (Molecular Probes). Nuclei were counterstained with DAPI according to manufacture protocol.

### Immunoblot Analysis

After stimulation with Wnt5a (1 μg/ml, 30 minutes, 1 hour, 2 hours), Gö 6976 (100 nM, 30 minutes), and/or rottlerin (1 μM), EPC was lysed with lysis buffer (Cell Signaling) containing 1 mM phenylmethanesulfonyl fluoride. After centrifugation the supernatants were collected and subjected to electrophoresis in 10% SDS-polyacrylamide gels. Proteins were transferred to poly vinylidene difluorid membrane, and incubated with anti-PKC delta (Cell signaling), anti-phospho PKC delta (Thr505, Cell Signaling), anti-phospho PKC alpha/beta2 (Thr638/641, Cell Signaling), anti-PKC alpha (Cell signalling), anti-PKC beta2 (Santa Cruz), or anti-alpha tubulin (Dianova) overnight at 4°C. Bound antibodies was visualized by using horseradish peroxidase (HRP)-conjugated sheep anti-mouse or donkey anti-rabbit antibody (both Amersham).

### Preparation of Triton-soluble and Triton-insoluble fractions

Cell pellets were resuspended in 1.0% Triton X-100, 50 mM HEPES, pH 7.4, 5 mM EGTA, 5 mM MgCl_2_, 30% glycerol, 0.5 mM dithiothreitol (DTT), 100 mM NaCl, and Complete Protease Inhibitor Cocktail Tablet (Roche), and incubated on ice for 20 min. After centrifugation (15,000G, 15 min.), the top fraction was collected as the noncytoskeletal (Triton-soluble, TS) fraction. The cytoskeletal (Triton-insoluble, TI) fraction was isolated by sonication (output 5×5 s) in the same buffer without Triton X-100.

### RNA isolation and RT-PCR analyses

Total RNAs from EPCs and rat CM were isolated by using TRIzol (Invitrogen). RNA was subjected to RT-PCR by using SuperScript First Strand Synthesis System (Invitrogen). Human specific primers were used as following: troponin T (5′-GAAGAAGAAGAGGAAGCAAAGGAG-3′/5′-TCCTTCTCCCGCTCATTCC-3′), GAPDH (5′-GAAGGTGAAGGTCGGAGTC-3′/5′-GAAGATGGTGATGGGATTTC-3′), alpha myosin heavy chain (5′-GTCCCGGCAGCTAGAGGA-3′/5′-CCTCTGTCTCCTCCTCGTAC-3′).

### Statistical analysis

Data are expressed as mean±SEM. Unpaired two-tailed students t-test (to compare 2 groups) or ANOVA (≥3 groups) were used for the comparison between groups based on the original data.

## Supporting Information

Figure S1 (2.74 MB TIF)
