# Supplementary figures and images for: Wnt5a Increases Cardiac Gene Expressions of Cultured Human Circulating Progenitor Cells via a PKC Delta Activation
Source: PLoS One. 2009 Jun 2;4(6):e5765. doi: 10.1371/journal.pone.0005765 (PMC2686162; doi:10.1371/journal.pone.0005765)

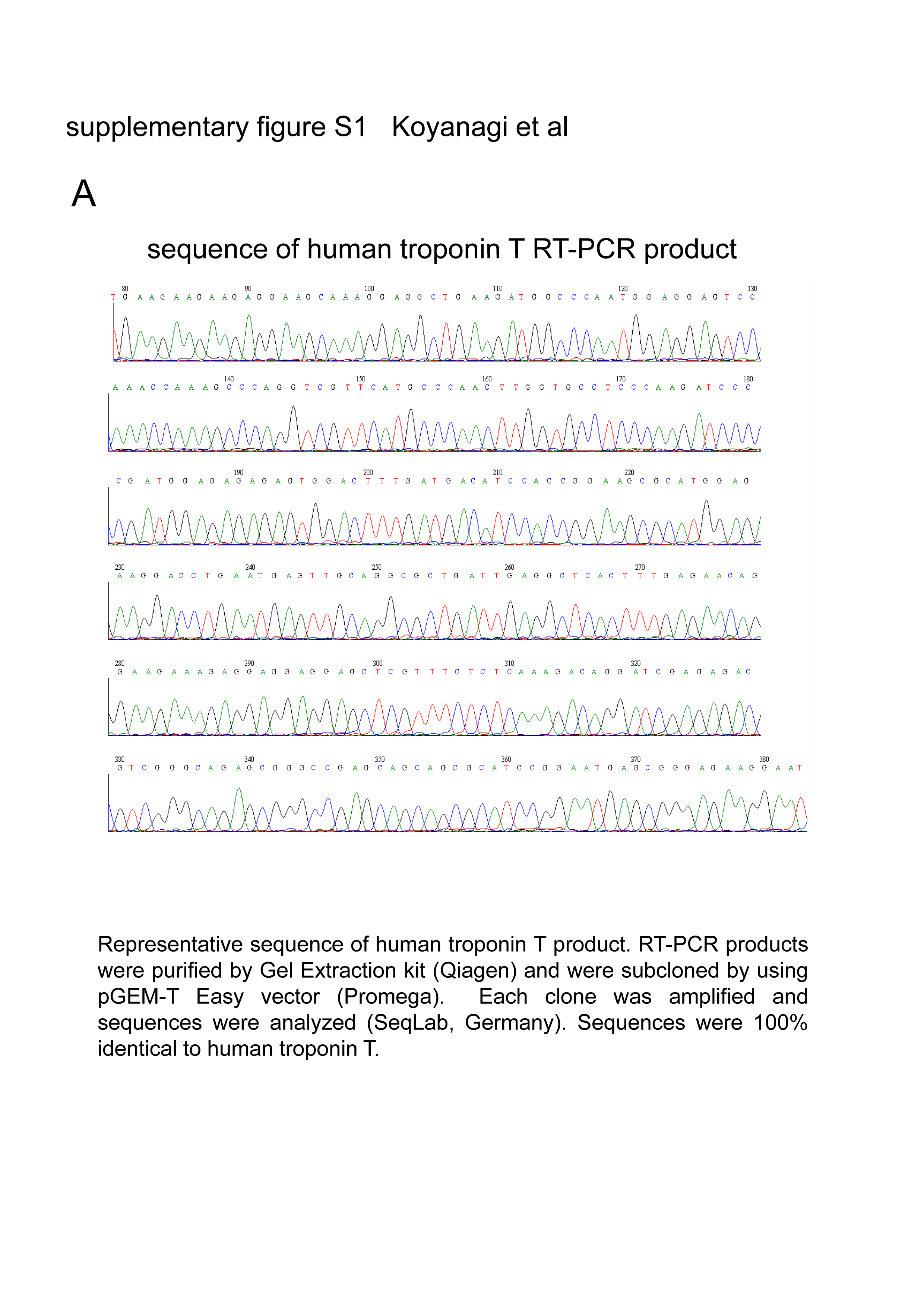

Supplement: Figure S1 — (2.74 MB TIF) [file pone.0005765.s001.tif]
